# Revising diagnosis of juvenile idiopathic arthritis in adults: a single-center retrospective study

**DOI:** 10.1007/s00296-023-05293-7

**Published:** 2023-03-01

**Authors:** Anna Felis-Giemza, Kornelia Chmurzyńska, Beata Kołodziejczyk, Agnieszka Gazda

**Affiliations:** 1 Biologic Therapy Center, National Institute of Geriatrics, Rheumatology and Rehabilitation, Warsaw, Poland; 2 Clinic and Polyclinic of Systemic Connective Tissue Diseases, National Institute of Geriatrics, Rheumatology and Rehabilitation, Spartańska 1, 02-637 Warsaw, Poland; 3 Clinic and Polyclinic of Rheumatology of Developmental Age, National Institute of Geriatrics, Rheumatology and Rehabilitation, Warsaw, Poland

**Keywords:** Arthritis, Juvenile, Tumor necrosis factor inhibitors

## Abstract

The study aimed to assess how many adult patients with juvenile idiopathic arthritis (JIA) treated with biologics fulfill classification criteria for adult rheumatic diseases and to evaluate the course of JIA in adulthood. 138 patients with JIA over 18 years old treated with biologics were included in a cross-sectional observative study. Among 138 adult patients with JIA treated with biologics, 81 patients remained with JIA diagnosis. 57 patients were rediagnosed. 31 patients met the criteria for spondyloarthropathy, among them 18 patients for ankylosing spondylitis, 10 patients for psoriatic arthritis, and 3 patients for non-radiographic axial spondyloarthritis. Rheumatoid arthritis was diagnosed in 24 patients and adults’ Still disease in 2 patients. 84 patients of all adults with JIA received one biologic agent, 40 received two biologic agents, and 14 received three or more biologic therapies. 10 patients received biologic agents out of recommendations for JIA. Of the adult JIA patients treated with biologics, 41% met the classification criteria for adult inflammatory diseases. Spondyloarthropathy and rheumatoid arthritis were most commonly diagnosed. Nearly 40% of adult JIA patients required at least one modification of biological treatment. Therefore, it is worth considering a revision of JIA to adult-onset inflammatory disease entities, as it broadens the spectrum of disease-modifying drugs.

## Introduction

Juvenile idiopathic arthritis (JIA) is a group of arthropathies with onset before age 16, with varied symptomatology. The International League of Associations for Rheumatology (ILAR) classification distinguishes seven types of JIA [1]. No type of JIA shares the exact name of adult arthritis.

Nomenclature allows us to choose therapeutic algorithms and determines reimbursement. The treatment of adult-onset arthritis is carried out with a broader spectrum of drugs than childhood-onset inflammatory diseases. In addition, more is known about adult-onset entities in terms of management and family planning.

The decision to implement biologic therapy is based on an assessment of disease activity and the presence of risk factors [2]. In JIA biologic treatment often starts in childhood and continues into adulthood. Sometimes the drug must be discontinued due to ineffectiveness or adverse event. If an adult patient with JIA meets the classification criteria of adult-onset arthritis, there is a possibility to change the drug to an agent registered and reimbursed for the treatment of this entity.

The retention rates for the first biologic agent in JIA are high throughout 4 years [3], but more than half of patients discontinue treatment with the first biologic drug, mainly because of ineffectiveness [4]. Thus, the limited number of biologic drugs approved for JIA results in searching for solutions elsewhere.

This study aimed to assess how many adult patients with JIA treated with biologics fulfill classification criteria for adult rheumatic diseases and to evaluate the course of JIA in adults treated with biologics after reaching adulthood.

## Materials and methods

This cross-sectional observative study took place at the Biological Therapy Center of the National Geriatrics, Rheumatology and Rehabilitation Institute in Warsaw. Between January and October 2021, we recruited patients from the biologic treatment registry over the age of 18, with JIA of at least 4 years duration, treated between the years 2010–2021. Biologic therapy was defined as taking inhibitors of tumor necrosis factor-alpha, interleukin-6, interleukin-17, and Janus kinase. Data were collected retrospectively from available pediatric and adult medical records from biologic treatment registries, outpatient clinic, and hospitalizations. They included demographic information, the form of JIA, the treatment used, adult diagnosis, the finding of ever-onset anti-citrullinated protein antibodies (ACPA), rheumatoid factor (RF), HLA-B27 antigen and sacroiliitis on imaging (at least grade I unilateral on magnetic resonance tomogram or radiograph based on radiologist opinion, any time in life).

The patients were rediagnosed by the clinician rheumatologists in course of their treatment. The rediagnosis was made if the adult patient with JIA met one of the following criteria: 2010 American College of Rheumatology (ACR)/European Alliance of Associations for Rheumatology rheumatoid arthritis (RA) criteria, 1987 ACR RA criteria, modified New York criteria for ankylosing spondylitis (AS), 2009 Assessment of Spondyloarthritis International Society (ASAS) for axial spondyloarthropathy, CASPAR criteria or Yamaguchi diagnostic criteria for adult-onset Still disease (ASD).

The efficacy of the treatment had been monitored at 3- to 6-month intervals using disease-specific activity scores. The drug was continued, if at the third month improvement occurred and in the following visits patient achieved low disease activity or remission.

The main outcome was the percentage of patients with JIA who meet the criteria for adult-onset arthritis and the number of biologic agents used by patients with JIA who reached adulthood.

Characteristics of JIA patients and statistical significance were performed using the chi-squared test, Fisher’s exact test, or *t* test. For all analyses, differences were considered statistically significant when the *p* value was < 0.05. For the management of missing data pairwise deletion was used.

The study complied with the ethical standards in accordance with Helsinki Declaration and national regulations in the field. Ethical approval was not required as this was a non-interventional, retrospective database study based on data collected in course of treatment.

## Results

A total of 138 adult patients with a JIA treated with biologics were recruited for the study. Detailed demographic and clinical features are shown in Table 1.

**Table 1:** Adults with juvenile idiopathic arthritis treated with biologic agents. Patients’ characteristics

JIA subtype	Number *n* (%)	Sex f/m	HLA-B27 number/tested (%)	Sacroiliitis number/tested (%)	RF number/tested (%)	ACPA number/tested (%)
All	138 (100%)	88/50	36/70 (51%)	33/61 (54%)	10/122 (8%)	10\96 (10%)
Systemic	10 (7%)	4/6	0/5	0/3	0/7	0/7
Oligoarhtritis	23 (17%)	19/4*	4/8 (50%)	2/7 (29%)	0/17	0/11
Persistent	9	8/1	3/5 (60%)	1/4 (25%)	0/7	0/3
Extended	13	10/3	1/3 (33%)	1/3 (33%)	0/10	0/8
No data	1	1/0	0/0	0/0	0/0	0/0
Polyarthritis	46 (33%)	41/5*	4/18 (22%)	2/10 (20%)	10/43 (23%)	9/39 (23%)*
RF+	10	10/0	0/2	0/1	10/10 (100%)	8/10 (80%)
RF−	36	31/5	4/16 (25%)	2/9 (22%)	0/33	1/29 (3%)
ERA	28 (20%)	7/21*	17/21 (81%)*	20/23 (87%)*	0/27	0/16
PsA	5 (4%)	4/1	1/4 (25%)	3/4 (75%)	0/5	0/4
Undifferentiated	10 (7%)	4/6	6/8 (75%)	4/9 (44%)	0/10	0/6
No data	16 (12%)	9/7	4/6 (67%)	2/5	0/13	1/13 (8%)

*ACPA* anti-citrullinated protein antibodies, *ERA* enthesitis-related arthritis, *JIA* juvenile idiopathic arthritis, *PsA* psoriatic arthritis, *RF* rheumatoid factor, *RF*+ rheumatoid factor positive, *RF−* rheumatoid factor negative

*p < 0.05

The largest number of patients were diagnosed with polyarthritis and enthesitis-related arthritis (ERA). Women were a majority in the general population (female-to-male ratio 1.76:1). The mean age of disease onset was 8.7 years.

The presence of the HLA-B27 antigen was detected in 51% of tested patients, most frequently in patients with ERA and undifferentiated JIA. Sacroiliitis on imaging was described in 54% of examined patients, most commonly in patients with ERA and psoriatic arthritis (PsA).

RF was detected in 8% of tested. RF positive (RF+) polyarthritis was diagnosed in all of them. ACPA was detected in 10% of examined patients. RF and ACPA were detected simultaneously in 8 patients.

The criteria for adult-onset arthritis were met by 57 patients. The remaining patients were left with a JIA diagnosis (Table 2). The most common diagnosis was spondyloarthritis (31 patients) and included AS (18 patients), PsA (10 patients), and non-radiographic axial spondyloarthritis (3 patients). RA was diagnosed in 24 patients, including 7 seropositive and 15 seronegative forms, with 2 missing data. ASD was diagnosed in 2 patients.

**Table 2:** Percentage of patients with juvenile idiopathic arthritis types fulfilling classification criteria for adult rheumatic diseases comparing to literature data^a,b^

	RA, *n* = 24	Spondyloarthritis, *n* = 21	PsA, *n* = 10	ASD, *n* = 2	Undifferentiated	Adults with JIA, *n* = 81
Systemic, *n* = 10	40% (5.1–25%)	0 (0)	0 (0)	0 (25–92.3%)	0 (2.6–6.3%)	60% (0)
Polyarthritis, *n* = 46	37%* (61–77%)	0 (4–11%)	6.5% (3–7%)	0 (0–3%)	0 (12–14%)	56.5% (0)
RF− polyarthritis, *n* = 36	28% (52–57.1%)	0 (7.6–16%)	8% (0–12.7%)	0 (0–4%)	0 (16–23.8%)	64% (0)
RF+ polyarthritis, *n* = 10	70%* (81.8–95.6%)	0 (0–1.5%)	0 (1.5–9.1%)	0 (0)	0 (1.5–9.1%)	30% (0)
Oligoarthritis, *n* = 23	0 (21–25%)	13% (26–27%)	9% (0–5%)	0 (0–2%)	0 (25–48%)	78%* (0)
Persistent oligoarthritis, *n* = 9	0 (6.1–25.8%)	11% (16.1–27%)	11% (0–7.6%)	0 (0–3.2%)	0 (32.2–59.1%)	78% (0)
Extended oligoarthritis, *n* = 13	0 (23.1–38.9%)	15% (24–53.8%)	8% (0–1.9%)	0 (0)	0 (15.4–35.2%)	77% (0)
ERA, *n* = 28	0 (0)	50%* (85–86.8%)	0 (0–7.9%)	0 (0)	0 (5.3–7.7%)	50% (0)
PsA, *n* = 5	0 (0)	0 (0)	80%* (92.3%)	0 (0)	0 (7.7%)	20% (0)
Undifferentiated, *n* = 10	0 (33.3–50%)	20% (16.7–33,3%)	10% (0)	0 (0)	0 (16.7–50%)	70% (0)
No data, *n* = 16	19%	12.5%	0	12.5%	0	56% (0)
All types, *n* = 138	17.4% (32.8–34%)	15.2% (24.4–31.1%)	7.2% (0.8–8.6%)	1.4% (4.9–9.4%)	0 (13.9–21%)	58.7% (0)

Spondyloarthritis includes ankylosing spondylitis, enteropathic arthritis and undifferentiated spondyloarthritis, excluding psoriatic arthritis

*ASD* adult-onset Still disease, *ERA* enthesitis-related arthritis, *JIA* juvenile idiopathic arthritis, *PsA* psoriatic arthritis, *RA* rheumatoid arthritis, *RF*+ rheumatoid factor positive, *RF−* rheumatoid factor negative

*p < 0.05

^a^Dzhus M (2017) Juvenile Idiopathic Arthritis in Adults: Long-Term Observation of Ukrainian Patients. Archive of Clinical Medicine. https://doi.org/10.21802/acm.2017.1.5

^b^Oliveira-Ramos F, Eusébio M, M Martins F et al. (2016) Juvenile idiopathic arthritis in adulthood: fulfillment of classification criteria for adult rheumatic diseases, long-term outcomes and predictors of inactive disease, functional status and damage. RMD Open. *2*(2) https://doi.org/10.1136/rmdopen-2016-000304

The time from onset to last visit was at mean 14 years (SD ± 6 years). The duration of the disease was at mean 15 years in rediagnosed patients and 13 years in patients who remained with JIA diagnosis (*p* > 0.05). Biologics were introduced at mean 7 years after disease onset (SD ± 5 years). The biologic therapy started at mean 8 years after onset in rediagnosed patients and 7 years after onset in the patients who remained with JIA diagnosis (*p* > 0.05).

One biologic drug was prescribed to 84 patients, and the second biologic drug was prescribed to 40 patients. Three or more lines of treatment were prescribed to 14 patients, including 6 with PsA. For 93 patients, the first drug was etanercept (ETA), for 39 it was adalimumab (ADA), and for 6 it was tocilizumab (TOCI).

In 10 patients, including 5 with PsA, 3 with RA, and 2 with AS, medications outside the ACR recommendations for JIA were used [2], while complying with recommendations for adult diagnoses. In 8 patients, the reason was the ineffectiveness of previous treatment (after 8.4 years of biologic treatment at mean), in 1 an additional condition (uveitis), and in 1 pregnancy. Secukinumab was prescribed to 6 patients, certolizumab pegol (CZP) to 4 patients, tofacitinib to 2 patients, and ixekizumab to 1 patient. Two or more medications outside the ACR recommendations were prescribed to 2 patients.

## Discussion

In the era of biological treatment, adult patients with JIA can be treated with biologics for most of their lives, but studies on adult patients with JIA are scarce in the literature. In terms of JIA form and sex, the population in our study mirrored the distribution in the general population, except for a much lower prevalence of oligoarthritis (17% vs. 30–55%) [5–9]. In McErlane’s study of adults with JIA treated with biologics, the percentage was also 17%, which the authors associate with the natural course of oligoarthritis characterized by the most frequent occurrence of remission within 10 years of disease onset [9].

The prevalence of the HLA-B27 antigen among JIA patients ranges from 27 to 30.7% [6, 10, 11]. A higher percentage in our observation (51%) may be caused by a large number of patients with the ERA among the study subjects (30% of patients tested for HLA-B27). The HLA-B27 antigen is most prevalent in patients with the ERA (57.7–97%) and undifferentiated forms (86.7%) [7, 8, 11, 12], which we also observed among our patients. Another factor contributing to the high percentage of HLA-B27-positive patients in our study may be biologic treatment, as the presence of this antigen correlates with the ineffectiveness of disease-modifying drugs [11] and higher disease activity [12]. It is also noteworthy that 6 of the 7 patients with spondyloarthritis, requiring treatment outside the ACR recommendations for JIA, had the HLA-B27 antigen present (1 was not tested).

Sacroiliitis in ERA occurs in 16–55.6% of patients [8, 12, 13] but may be clinically silent in 1/5 of patients [13]. Of note is the nearly twofold higher percentage of patients with sacroiliitis in our observation (87%). This may be due to the selection of the group of patients treated with biologics—patients with sacroiliitis achieve remission less often than other patients with ERA [8].

The prevalence of RF and ACPA increases with age. RF is present in 24.6–27.5% of adult patients with JIA and only 4% of children with JIA [6, 7, 10]. ACPA is present in 2.8% of children [7] and 10–30.8% [6, 10] of adults with JIA. The presence of ACPA or RF is a factor in poor prognosis and should be considered in therapeutic decision-making [2]. Despite this, RF and ACPA were not more frequent in the patients in our observation than in the reported sources.

The studies of Oliveira-Ramos and Dzhus [6, 10] and our observations allow us to predict the directions in which the different forms of JIA will evolve (Table 2). Systemic arthritis mostly evolves into ASD or RA; polyarthritis into RA, and to a lesser extent into PsA. The RF negative (RF-) form less frequently transforms into RA. Patients with PsA most often remain with this diagnosis (CASPAR and ILAR criteria for PsA are similar, but not identical). ERA evolves into spondyloarthritis. The most diverse course can be observed in oligoarthritis, which can evolve into any type of arthritis. Moreover, among all subtypes of JIA, up to 1/5 of patients are diagnosed with undifferentiated arthritis in adulthood. In our observation, such patients remained in the group of patients with a persistent diagnosis of JIA, which was the most significant difference from the sources mentioned.

Nigrovic et al. [14] advocate a departure from previous classifications. Instead, they propose distinguishing four types common to all age groups, based on related genetic background: RF+ arthritis, RF− arthritis, spondyloarthritis, and systemic arthritis. In this sense, JIA does not develop into adult-onset arthritis but follows a pathway traced by genetic determinants from the beginning.

Compared to the McErlane study [9], more patients used only one biologic drug (61% vs. 50%), and fewer required three or more lines of treatment (10% vs. 22%).

At the time of the implementation of CZP in our patients, the 2019 ACR recommendations noted that CZP was not included due to a lack of data for the pediatric population. This example shows that a drug effective in adult-onset disease has appeared late in pediatric recommendations. That affects the therapy of adults with the childhood-onset disease. Echoing McErlane and Feger, we point to the lack of consensus on therapy for adults with JIA [9, 15].

Our study had a few limitations. Children with JIA treated with classic DMARDs diffuse into various centers after reaching adulthood and were not included in the study. Some of the data were unavailable, which reduced the size of individual groups. In addition, the data came from clinical practice: investigations were targeted, not routine. It is not possible to compare the drugs used with the literature, because treatment centers differ in their choice of drugs (at our center, ETA, ADA, and TOCI only were available for both children and adults with JIA).

## Conclusions

The adult population with JIA is highly heterogeneous. Of the JIA patients treated with biologics, less than half of the patients met the classification criteria for adult inflammatory diseases. In this group of adults with JIA, the most common diagnoses were spondyloarthritis and RA. Nearly 40% of adult JIA patients treated with biologics required at least one modification of biologic treatment. More than one modification of biologic treatment was required by 10% of patients, most often meeting the classification criteria for PsA.

Therefore, it is worth considering a revision of JIA to adult-onset inflammatory disease entities, as it broadens the spectrum of disease-modifying drugs.

## Data Availability

The statistic data that support the findings of this study are available from the corresponding author upon reasonable request.
